# To the International Hahnemannian Association

**Published:** 1893-03

**Authors:** Overton F. MacDonald

**Affiliations:** Toronto


### TO THE INTERNATIONAL HAHNEMANNIAN ASSOCIATION.

Toronto, June 15th, 1892.

Mr. B. came to me on April 5th, 1891, with a very psoric constitution, and suffering from overwork. He had contracted scabies shortly before coming.

He was told that the eruption must be left alone, that no local treatment must be resorted to; and a remedy was selected in accordance with his constitutional condition. This proved to be Sulphur$^{cm}$.

His general health improved considerably, and some months later the case ran into Sepia, for which he got the 50m.

He remained under treatment until he went to England in August.

Though improved greatly in general health, the eruption had got steadily worse, apparently without being in any way affected by the treatment. He was in a dreadful state with it when he went to England.

During his stay in Canada, previous to his going to England, he had visited several places in various parts of the country; his business compelling him to do so.

Wherever he went, he seemed to leave the disease behind him. I heard of half a dozen cases in different families contracted from him.

Three or four cases came to me for treatment.

Of these only one remained under my care for any length of time. They were all treated with the homœopathic remedy only, and great care bestowed upon its selection. None were benefited in the slightest degree.

The one case that remained was that of young lady.

Mrs. B. had visited at her father's house.

Her father was a patient of mine as were also several members of her family.

She remained under treatment for six months, closely following the directions.

The remedies in her case were:

Silicea$^{40m}$, F.

Sulphur$^{21m}$.

Sepia$^{50m}$.

All selected for constitutional indications.

Her general health was improved, but the eruption got steadily worse; the remedies having no effect upon it.

At last in despair I gave Alumina$^{50m}$, F. C., which had done grand work in another member of her family, and for which there were some indications in her case.

There was no improvement in the rash.

The girl was suffering dreadfully from the irritation, and her friends would not come near her, being afraid of contagion. She was practically quarantined by all who knew her.

Her father was an old patient of Dr. Constantine Hering, and had great faith in everything the doctor had ever said or done. He pointed out to me, that in his work on domestic treatment, Dr. Hering advises the killing of the itch bug by Sulphur ointment, saying it will not injure the patient; and, at last, all other means having failed, and the girl's condition getting worse and worse, I consulted several physicians who are members of the International Hahnemannian Association.

After thinking it over, the doctors agreed with me that, the remedies having failed, I should use Sulphur locally and give her relief. I explained to them that it was not homœopathic treatment and told them to use it.

A few days ago I wrote the father for an account of the case, and append extracts from his letter. He says: "You know I am not an enemy of Homœopathy, but it seems to me to be *practically* a failure in acarus itch. Who is going to put up with six months or more such suffering as it is, when a few applications of Sulphur ointment will settle the matter?

"Dr. —— (mentioning the name of one of the most successful Hahnemannians in Canada) has not been a whit more successful than yourself in the cases that he had in hand as far as I know. I shall be glad to know what the International Hahnemannian Association says about it, and am pleased that you are going to lay the matter before the Association. My daughter is now well."

A number of years ago, the greatest prescriber Canada has ever had in Homœopathy had one of Toronto's public institutions under his care when scabies broke out. In spite of everything he could do, the contagion ran through the institution until the Board of Management interfered and called in allopathic advice. The allopaths stamped the disease out in a few days, and no homœopath has ever been allowed to treat a case in that institution since.

It does seem as if scabies were not a case for medical treatment but for a direct poison for the bug.

Dr. E. T. Adams has kindly consented to bring the matter before the Association as I cannot attend the meeting this year.

Trusting the International Hahnemannian Association will give this their consideration, I remain,

Respectfully,

OVERTON F. MACDONALD, M. D.

